# Case report: Acute right ventricular dysfunction after surgery in a pregnant patient with congenital heart disease and aortic dissection

**DOI:** 10.3389/fcvm.2023.1146158

**Published:** 2023-03-23

**Authors:** Junhai Hao, Siyi Liu, Tucheng Sun, Liming Lei

**Affiliations:** ^1^ Department of Intensive Care Unit of Cardiac Surgery, Guangdong Cardiovascular Institute, Guangdong Provincial People's Hospital (Guangdong Academy of Medical Sciences), Southern Medical University, Guangzhou, China; ^2^ Laboratory of South China Structural Heart Disease, Guangzhou, China; ^3^ Department of Cardiac Surgery, Guangdong Cardiovascular Institute, Guangdong Provincial People's Hospital (Guangdong Academy of Medical Sciences), Southern Medical University, Guangzhou, China

**Keywords:** acute right ventricular dysfunction, cardiac surgery, aortic dissection, pregnant patient, congenital heart disease

## Abstract

Pregnant women with aortic dissection are hemodynamically outmost complex patients. The two major diagnoses that should be considered in pregnant patients with congenital heart disease (CHD) and acute type A aortic dissection presenting with postoperative right ventricular dysfunction are pulmonary thromboembolism and right ventricular infarction. We present a rare case of postoperative right ventricular dysfunction in pregnant women with CHD and acute aortic dissection, which was diagnosed by pulmonary computed tomography angiography and treated by percutaneous coronary intervention.

## 1. Introduction

**Figure 1:**
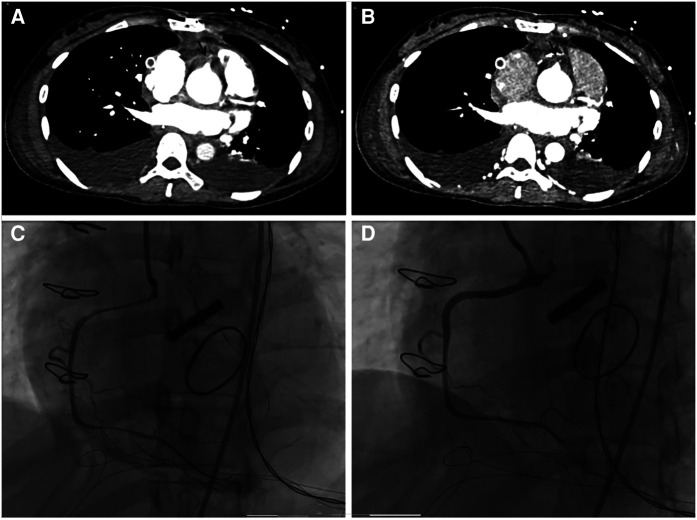
(**A,B**) pulmonary artery computed tomography angiography showed occlusion of the initial of the right coronary artery (black arrow). (**C**) Coronary angiography revealed occlusion of the right coronary artery (RCA) ostium, which was significantly improved after percutaneous coronary intervention (**D**).

**Figure 2:**
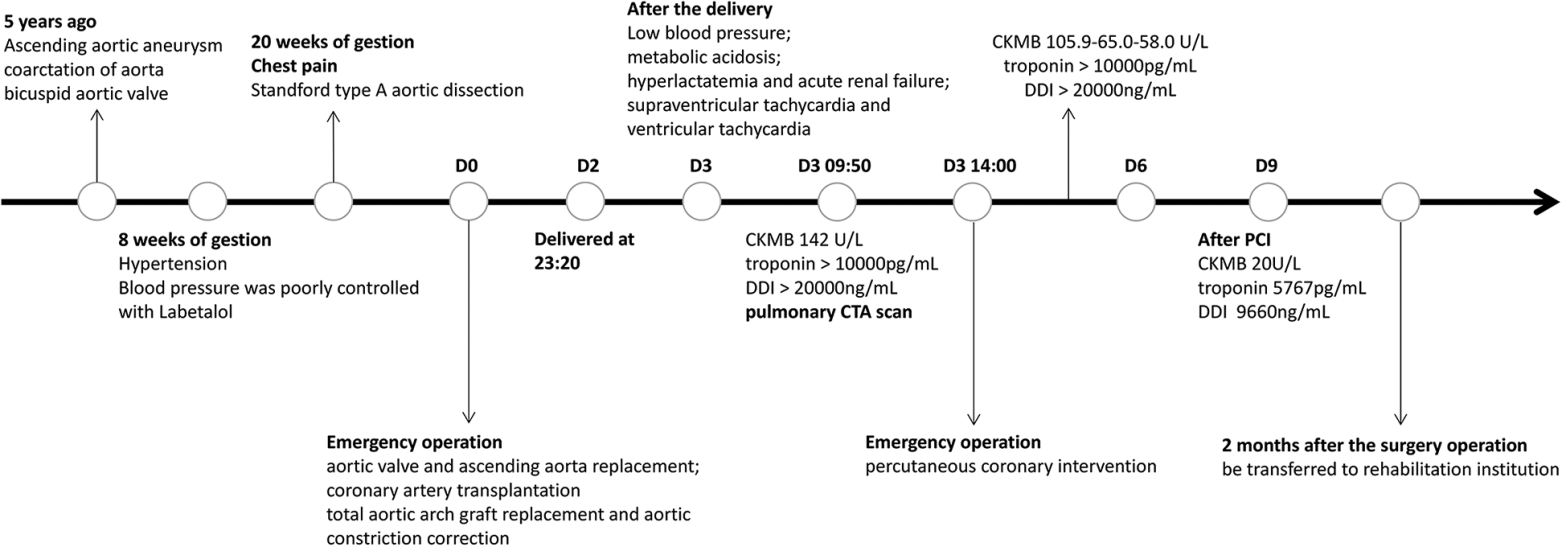
Timeline.

A 30-year-old pregnant woman with a previous diagnosis of an ascending aortic aneurysm (diameter of the ascending aorta: 71 mm) and a history of congenital heart disease (coarctation of the aorta (CoA), bicuspid aortic valve (BAV)) presented with a severely elevated blood pressure of 170/80 mmHg at 8 weeks of gestation. Before this, she had been aware of the risk that ascending aortic aneurysm secondary to BAV and CoA malformations could lead to aortic dissection, but she still refused the advice of surgical treatment. The patient had poorly controlled blood pressure (120–170/80–90 mmHg), despite receiving labetalol (75 mg twice a day). At 20 weeks of pregnancy, she experienced severe recurrent chest pain. Computed tomography angiography (CTA) scan of the aorta revealed “Stanford type A aortic dissection and aortic coarctation. Ascending aortic aneurysmal dilatation with a maximum diameter of 7 cm. An intimal tear was observed in the noncoronary aortic sinus, localized dissection was formed. Significant dilatation of the sinotubular junction. The left and right coronary arteries originated from the true lumen and showed no significant stenosis”. The echocardiography scan demonstrated BAV with mild regurgitation (regurgitation area of 1.5 cm^2^) and left ventricular ejection fraction (LVEF) of 60%. Due to the high associated risk of mortality, the patient received emergent surgery after signing an informed consent form. She underwent aortic valve and ascending aorta replacement, coronary artery transplantation, total aortic arch graft replacement, and aortic constriction correction. Intraoperative recordings showed that cardiopulmonary bypass time was 351 min, aortic cross-clamp time 227 min, selective cerebral perfusion time 43 min, mean pressure maintained at 80–90 mmHg, and perfusion flow 2.8–3.6 L/(min*m^2^). On the first post-operative day, the patient was hemodynamically stable, with a normal blood lactate level, negative troponin test, and a creatine kinase myocardial band (CKMB) of <10 U/L. After the mechanical valve replacement (Bentall procedure), intravenous heparin injection was started on D2, and heparin dosage was adjusted according to activated partial thromboplastin time (APTT). Obstetric ultrasound scans demonstrated “intrauterine fetal death”. On the second day, the patient delivered a stillborn fetus with intact placental membranes. She experienced approximately 200 ml of blood loss during the delivery. After the delivery, she experienced a rapid drop in her blood pressure to 85/40 mmHg and developed metabolic acidosis, hyperlactatemia, and oliguric acute renal failure. She had elevated serum creatinine (335.8 µmol/L) and bilirubin (131.2 µmol/L) levels. High-dose vasoactive drugs (epinephrine 0.1 µg/kg/min and dobutamine 10 µg/kg/min) were required to maintain her blood pressure. This patient also experienced several episodes of supraventricular tachycardia (heart rate 160 beats/min) as well as episodes of vantricular tachycardia (heart rate 180–220 beats/min). Echocardiography scans demonstrated a left ventricular cavity diameter of 35 mm, an estimated LVEF of 45%, a right ventricular end-diastolic diameter (RVEDD) of 50 mm, and an estimated right ventricular LVEF of 30% (bedside simple echocardiography, left ventricular long-axis view and apical four-chamber view). Electrocardiography suggested an axis of 120° (remarkable right deviation), inverted T waves in leads II, III, and aVF, and complete right bundle branch block (CRBBB). The patient's serum CKMB increased to 142.0 U/L. Her troponin and D-dimer levels were measured to be >10,000 pg/ml and >20,000 ng/ml respectively. As the patient had a recent history of delivery, the possible diagnoses included amniotic fluid embolism, acute pulmonary thromboembolism, and acute myocardial infarction. The patient underwent an immediate pulmonary artery CTA scan that demonstrated no obvious signs of thrombus in the main trunk or its branches. However, there was an occlusion of the origin of the right coronary artery and myocardial ischemia of the lower wall of the base and middle of the left ventricle (Figures 1A,B). Acute myocardial infarction was clinically diagnosed based on these findings, the elevated serum CKMB, and the new regional wall motion abnormality seen on echocardiography scans. Urgent coronary angiography was performed, followed by percutaneous transluminal coronary angioplasty (PTCA) and stent implantation. Angiography demonstrated occlusion of the right coronary artery (RCA) ostium. Dynamic re-examination revealed a slow decrease in CKMB (105.9 to 65.0 to 58.0 U/L) while her troponin and D-dimer levels remained >10,000 pg/ml, and >20,000 ng/ml respectively for 3 days (D4–D6). The patient's serum CKMB, troponin, and D-dimer levels gradually decreased after percutaneous coronary intervention (PCI) to 20 U/L, 5,767 pg/ml, and 9660 ng/ml respectively on the 9th-day post-procedure. Aspirin combined with warfarin, which replaced the intravenous heparin injection was started on D10. Re-examination of the echocardiography demonstrated an end-diastolic diameter of 42 mm, LVEF of 54%, coordinated myocardial contraction, and a mild tricuspid valve (bedside simple echocardiography, left ventricular long-axis view, and apical four-chamber view). The patient also experienced other complications, such as pulmonary infection and hypernatremia during her hospitalization. She was treated with mechanical ventilation, antibiotics, nutritional support, and continuous renal replacement therapy (CRRT) for approximately 2 months. She was transferred to a rehabilitation institution for further treatment and safely discharged after 1 month. The figure below shows a timeline of the patient's medical history (Figure 2).

## 2. Discussion

The term “pregnancy complicated by cardiovascular disease” is used to refer to pregnant women who either have a history of or are suffering from emerging cardiovascular disease. The incidence of cardiovascular disease complicating pregnancy increases year by year with increasing maternal age, ranging from 1% to 4%. It is associated with a high mortality rate (1). The blood volume increases in pregnant women, peaking in the second trimester. This could lead to serious adverse cardiac events such as pulmonary edema and heart failure for pregnant women with cardiovascular disease (2). Current guidelines utilize the modified World Health Organization (mWHO) risk classification (mWHO grade–I–IV) to assess the risk of cardiovascular disease (CVD) in pregnant women with cardiovascular disease (1, 3). Pregnant women with mWHO grade III–IV have a significantly increased risk of complications and mortality and usually require cesarean sections. Women diagnosed with pulmonary hypertension, severe ventricular dysfunction (LVEF less than 30%, NYHA class III–IV), severe left ventricular outflow tract obstruction, severe thoracic aortic dilatation, and uncorrected aortic coarctation, etc., are classified as grade IV. They are advised to avoid pregnancy. If they insist on continuing the pregnancy, they should be made fully aware of the high risk of complications and mortality. During pregnancy, close monitoring of the mother and the fetus is required.

BAV is the most common congenital cardiac abnormality with an estimated prevalence of 1%–2%. Though most often occurring in isolation, BAV may be accompanied by other cardiovascular malformations, such as CoA (50%–80%), interruption of the aorta (36%), atrial or ventricular septal defects (20%) and anomalous coronary arteries (14%) (4, 5). According to the literature, aortic coarctation may be the most common associated cardiovascular malformation in BAV disease. The abnormal architecture of the valve makes the leaflets susceptible to haemodynamic stress, leading to valvular thickening, calcification, and increased rigidity and narrowing of the aortic orifice (5). Even if this lesion is clinically silent in early life, BAV can lead to cardiovascular sequelae, including aortic valve dysfunction and ascending aortopathy (aortic aneurysm and aortic dissection) (4). The incidence of aortic dissection in pregnant patients ranges from 0.1% to 0.3%. Hypertension, connective tissue disorders, and congenital heart disease especially BAV are well-known risk factors (6). Kreibich et al. reported the characteristics of aortic dissection in patients with BAV. They found that the aortic dissection in patients with BAV was significantly younger and performed with aortic root replacement more often than in patients with tricuspid aortic valves (TAV). Type A aortic dissection in BAV patients is not associated with worse short or long-term outcomes but significantly influences the proximal aortic repair (7). In the present case, the patient had ascending aortic aneurysm, CoA and BAV malformation without anomalous coronary arteries, and she also had poorly controlled hypertension, so this patient has a high risk of developing aortic dissection. With the mWHO classification of grade IV and the high risk of mortality, termination of pregnancy was recommended. The patient refused to terminate and developed aortic dissection later on.

Aortic dissection during pregnancy occurs most commonly in the third trimester, owing to the hyperdynamic state and hormonal effects on the vasculature during this period (3). It presents with sudden severe tearing, chest pain, vomiting, and syncope, mostly due to an acute pericardial tamponade. The treatment of aortic dissection during pregnancy is mainly dependent on the gestational age of the fetus, hemodynamic changes, and symptoms of organ ischemia. Zeebregts et al. (8) recommended that pregnant women undergo cesarean section and aortic surgery simultaneously after 32 weeks of gestation. Aortic dissection repair or replacement is recommended before 28 weeks of gestation, for fetal protection. The mode of treatment between 28 and 32 weeks of gestation is decided according to the effect of the condition on the mother and fetus. Patients with Stanford type A aortic dissection experience an acute onset and often require emergency aortic repair, whereas patients with type B aortic dissection can be treated medically if there is no bleeding or perfusion disorder of the main branch. Our patient progressed to type A aortic dissection at 20 weeks of gestation; therefore, emergency surgery for aortic dissection was performed. Unfortunately, intrauterine fetal death was confirmed after the surgery, and the pregnancy was terminated.

Prompt surgical treatment results in reduced maternal mortality due to aortic dissection (9, 10). Pregnant patients with a definite diagnosis of Stanford type A aortic dissection require urgent aortic surgery regardless of the gestational age of the fetus, while concomitant cesarean delivery depends on the fetal gestational age and the patient's expectations. Emergent aortic surgery with fetal preservation has been suggested when the gestational age is less than 28 weeks; continuation of pregnancy or induction of labor is decided according to whether the fetus is still alive after surgery. Cesarean section has been recommended when gestational age >28 weeks, immediately followed by aortic surgery after fetal delivery (11). However, pregnant women undergoing aortic repair or replacement with extracorporeal circulation have a fetal mortality rate of 36% (12). This could be attributed to the adverse effects of deep hypothermic cardiopulmonary bypass on the fetus. It could lead to abortion, premature delivery, fetal distress, stillbirth, fetal growth restriction, and significantly increase postoperative fetal mortality (13, 14). Therefore, the European Society of Cardiology recommended that shortening cardiopulmonary bypass times, maintaining perfusion flow >2.5 L/(min*m^2^), perfusion pressure >70 mmHg, maternal hematocrit >0.28, pulsatile perfusion, and normothermic perfusion may improve fetal outcomes (13).

Sun et al. reported the surgical outcomes of 803 Stanford type A aortic dissection cases, with a total operative mortality of 6.5%. The incidences of respiratory complications, renal failure, thoracotomy hemostasis, spinal cord injury, and stroke were 15.57%, 3.4%, 3.1%, 2.4%, and 2.0%, respectively. However, postoperative acute myocardial infarction was not reported (15). Waterford et al. (16) reported that 38 of 1,445 patients (2.6%) with type A aortic dissection who underwent surgery developed acute myocardial infarction after surgery. There are limited data regarding postoperative acute myocardial infarction in pregnant women with acute type A aortic dissection.

Acute myocardial infarction (AMI) during pregnancy is an important cause of maternal death. A study observed a significant increase in the incidence of AMI during pregnancy in the United States from 2003 to 2015 (15). Hypertension, coronary artery disease, heart failure, valve replacement, atrial fibrillation, and traditional coronary atherosclerotic risk factors (hyperlipidemia, obesity, tobacco history, drug abuse, and thrombosis) were also discovered to be predictors and risk factors for AMI development in pregnant patients (17). A hypercoagulable state, aggravated heart disease, and hemodynamic disorders in pregnant women could also lead to pulmonary thromboembolism (18). In this case, the patient had long-term poorly controlled blood pressure, aortic valve disease, and a history of recent valve surgery. She was prone to an AMI. Aortic dissection intimal tears could obstruct the initial segment of the coronary artery. A hypercoagulable state and vasospasm could affect coronary artery patency after transplantation, leading to myocardial ischemia. After surgery, mechanical ventilation and sedative and analgesic drugs limit the expression of severe chest pain. This patient developed acute right ventricular dysfunction, coagulation disorders, and elevated D-dimer levels after the delivery. Therefore, we focused on differentiating between amniotic fluid embolism, acute pulmonary embolism, and AMI. The patient developed AMI without significant postpartum hemorrhage or significant bleeding and coagulation disorders, therefore, other diseases causing acute right heart failure need to be considered first. We identified right coronary artery occlusion and right ventricular infarction based on a pulmonary artery CTA scan. Hence, all scans should be thoroughly performed as a pulmonary artery CTA scan could also provide information about coronary arteries and myocardial infarction.

In conclusion, the delivery plan and peripartum management in women with significant cardiovascular risk factors and mWHO classification of class III and IV require specific cardiovascular recommendations or support. Pregnant women with aortic dissection are hemodynamically outmost complex patients. More attention should be given to changes in cardiac function after surgery in such patients. The two major diagnoses that should be considered in pregnant patients with acute type A aortic dissection presenting with postoperative right ventricular dysfunction are pulmonary thromboembolism and right ventricular infarction. It should be noted that a pulmonary artery CTA scan can also provide information about coronary arteries and myocardial infarction, hence, a careful reading of the scan is critical.

## Data Availability

The original contributions presented in the study are included in the article/Supplementary Material, further inquiries can be directed to the corresponding author/s.
